# Measuring clinically significant outcomes - LDQ, CORE-10 and SSQ as dimension measures of addiction

**DOI:** 10.1192/pb.bp.112.041301

**Published:** 2014-06

**Authors:** Duncan Raistrick, Gillian Tober, Jenny Sweetman, Sally Unsworth, Helen Crosby, Tom Evans

**Affiliations:** 1 Leeds Addiction Unit; 2 University of Leeds

## Abstract

**Aims and method** To determine values for reliable change and clinically significant change for the Leeds Dependence Questionnaire (LDQ) and Social Satisfaction Questionnaire (SSQ). The performance of these two measures with the Clinical Outcomes in Routine Evaluation (CORE-10) as three dimension measures of addiction was then explored.

**Results** The reliable change statistic for both LDQ and SSQ was ⩾4; the cut-offs for clinically significant change were LDQ ⩽10 males, ⩽5 females, and SSQ ⩾16. There was no overlap of 95% CIs for means by gender between ‘well-functioning’ and pre- and post-treatment populations.

**Clinical implications** These data enable the measurement of clinically significant change using the LDQ and SSQ and add to the evidence for the performance of the LDQ, CORE-10 and SSQ as dimension measures of addiction. The CORE-10 and SSQ can be used as treatment outcome measures for mental health problems other than addiction.

Transparency of the outcomes achieved by services is one means of driving quality improvement;^1^ however, if outcomes are to influence policy and commissioning, then the measures used must be up to the task. The Health Technology Assessment by Fitzpatrick^2^ describes three types of outcome measure:

- generic measures are concerned with an overarching concept, in this case health
- dimension measures are about a particular aspect of health, in this case addiction
- specific condition measures are focused on a diagnostic category, for example anxiety or personality disorder.

A rational approach to assessing treatment outcome is to combine a judicious mix of generic, dimension and condition-specific scales, which must be robust as judged by a quality framework.^3^

Addiction is usually assessed across three key dimensions: dependence, psychological well-being and social well-being,^4^ which are reflected in the scale selection.^5^ The Leeds Dependence Questionnaire (LDQ) measures dependence on psychoactive substances;^6^ it is a 10-item scale derived from a psychological understanding of dependence, reflects ICD-10,^7^ and has extensive and independent validation.^8-12^ The Social Satisfaction Questionnaire (SSQ) measures satisfaction with social circumstances^13^ and is an 8-item scale adapted from the Social Problems Questionnaire.^14^ Clinical Outcomes in Routine Evaluation (CORE-10 version) is a 10-item scale which measures psychological well-being; its psychometric properties have been comprehensively investigated and reported.^15^ This paper reports on the psychometric properties of the LDQ and SSQ required to estimate clinically significant change^16,17^ and then applies these, with CORE-10, to a clinical sample.

## Method

### Well-functioning population

A ‘well-functioning’ sample is required to calculate a cut-off for clinically significant change. We set a timescale within which to recruit an opportunistic sample from the local health service and the local university. The target was to equal in number the clinical sample; in the event, 817 individuals were recruited. Participants were invited to take part in the study by email sent through their National Health Service (NHS) or university workplace (and so response rates are not known) with a link to submit responses to the LDQ and SSQ online. Potential participants were given information about the study and understood that taking part was voluntary. They were invited to provide an email address if they wished to be entered into a prize draw for £50 vouchers. The study data and the email addresses for the prize draw were entered into separate, unconnected databases. Procedures were negotiated with a local research ethics committee. When participants logged on to the survey, the first screen asked them to disclose their age, gender, marital status, employment status, alcohol use (self-identified as ‘abstainer’, ‘occasional drinker’ or ‘regular drinker’) and drug use (‘yes’ or ‘no’). On subsequent screens they completed the LDQ and SSQ. Completion of the LDQ and SSQ was considered valid with up to two missing values within each scale; missing values were scored as the mean of the completed scores.

**Table 1:** Demographic details for functional and clinical groups

	Functional population, *n* = 817		Clinical population, *n* = 653	
	Male	Female	Male	Female
Gender, %	25.1	74.9	52.4	47.6
Age, years: mean (s.d)	38.6 (8)	36.3 (11.4)	43.7 (10.8)	41.7 (11.2)

**Table 2:** Mean scores for the Leeds Dependence Questionnaire (LDQ) and Social Satisfaction Questionnaire (SSQ)

			Clinical population, *n* = 653			
	Functional population, *n* = 817		Male		Female	
	Male	Female	Pre-treatment	Post-treatment	Pre-treatment	Post-treatment
LDQ, mean (95% CI)	4.3 (3.5-5.0)	2.5 (2.2-2.8)	17.8 (16.9-18.7)	8.6 (7.7-9.5)	10.9 (9.8-12.0)	4.2 (3.6-4.9)
SSQ, mean (95% CI)	18.6 (16.9-20.3)	19.1 (18.4-19.8)	13.8 (13.2-14.4)	15.3 (14.7-15.9)	16.0 (15.4-16.6)	17.3 (16.7-17.9)

### Dysfunctional (clinical) population

A clinical sample of 653 records was extracted from the local specialist addiction service clinical database; records were selected if age, gender, pre-treatment and paired post-treatment LDQ, SSQ and CORE-10 scores were available. Fifty-six per cent were in their first treatment episode, 24% were in the second and 20% the third or more. Completion of the LDQ, CORE-10 and SSQ was considered valid with up to two missing values within each scale. Post-treatment records were obtained between 3 and 12 months. Leeds Dependence Questionnaire and SSQ values for the ‘well-functioning’ group, and CORE-10 values taken from the published data^15^ were used to determine whether reliable or significant change occurred pre- to post-treatment. We have mirrored the methodology applied to evaluating the psychometrics of CORE-10 for the LDQ and SSQ so as to harmonise the three as dimension measures of addiction.

## Results

In the ‘well-functioning’ sample, 6.6% described themselves as abstainers from alcohol and were excluded from further analysis; 52.0% described themselves as occasional drinkers and 41.4% as regular drinkers; 9.4% said they used illicit drugs. The age, gender and mean scores of the LDQ and SSQ are shown in Tables 1 and 2. The reliable change scores are LDQ 3.5 (rounded to 4) and SSQ 4.3 (rounded to 4). There are no overlaps in the confidence intervals for the means of the LDQ and SSQ in the ‘well-functioning’ and ‘dysfunctional’ (clinical) populations either pre- or post-treatment compared by gender.

The cut-off points for the probability of belonging to the ‘well-functioning’ group are: LDQ (lower score is better) male 9.8 (rounded to ⩽10), female 5.0 (rounded to ⩽5); SSQ (higher score is better) male 15.3, female 17.2 (rounded to ⩾16 for both genders). Table 3 presents the percentage of those in treatment meeting the four outcome categories: reliable deterioration, no change, reliable improvement, and reliable improvement with clinically significant change. We investigated the effects of age and treatment episode on the achievement of clinically significant improvement. For three age groups (<35, 35-49, ⩾50 years old) the proportions achieving clinically significant change were: LDQ 33.6%, 42.4%, 51.4%; CORE-10 24.6%, 26.5%, 33.3%; SSQ 17.0%, 26.4%, 20.1%. For episode categories (1st, 2nd, 3rd or more) the proportions achieving clinically significant change were: LDQ 44.0%, 35.5%, 46.6%; CORE-10 28.5%, 27.3%, 26.2%; SSQ 22.6%, 21.3%, 22.9%. The baseline and follow-up correlations are LDQ and CORE-10: 0.65 and 0.61; LDQ and SSQ: –0.39 and –0.30; CORE-10 and SSQ: –0.47 and –0.56.

## Discussion

Jacobson & Truax^16^ and Jacobson *et al*^17^ have described three statistical methods for determining whether change after an episode of treatment is clinically significant. They conceptualise the clinical population as ‘dysfunctional’ and the aim of treatment as bringing individuals within the bounds of a ‘well-functioning’ population. They describe the method for determining a cut-off score for a scale which defines whether an individual is more likely to belong to the ‘well-functioning’ rather than ‘dysfunctional’ population as the least arbitrary of the three proposed methods. For clinically significant change to occur, a second criterion - reliable change - which takes account of measurement error must also be achieved. The reliable change value is the minimum improvement post-treatment which yields a 90% probability that change is not due to variations in measurement. Reliable change values are calculated from a post-treatment population;^15^ clinically significant cut-off scores are calculated from both ‘well-functioning’ and pre-treatment populations.^15^

**Table 3:** Outcomes for dimension measures LDQ, CORE-10 and SSQ

		%											
		Reliable deterioration			No reliable change			Reliable improvement			Reliable and clinically significant improvement		
	*n*	LDQ	CORE-10	SSQ	LDQ	CORE-10	SSQ	LDQ	CORE-10	SSQ	LDQ	CORE-10	SSQ
Gender													
Male	270	4.8	4.8	14.7	25.9	46.0	54.2	69.3	49.2	31.8	50.4	26.8	23.7
Female	241	4.6	9.6	13.6	44.0	45.4	59.1	51.4	45.0	29.6	33.6	28.8	22.9
All	511	4.7	7.0	14.2	34.4	45.7	56.5	60.9	47.2	30.7	42.5	27.7	23.3
Substance													
Alcohol	319	3.4	3.1	11.5	22.9	38.4	57.5	73.7	58.5	31.0	51.4	34.7	23.9
Heroin	68	4.4	13.2	10.3	48.5	48.5	51.5	47.1	38.2	38.2	35.3	19.1	29.4
Methadone	49	0	12.2	24.0	93.9	75.5	62.0	6.1	12.2	14.0	4.1	12.2	12.0
Stimulants	22	9.1	13.6	9.1	45.5	54.5	59.1	45.5	31.8	31.8	27.3	13.6	22.7

CORE-10, Clinical Outcomes in Routine Evaluation; LDQ, Leeds Dependence Questionnaire; SSQ, Social Satisfaction Questionnaire.

A general population sample might be the ideal ‘well-functioning’ sample but recruitment would be difficult and costly. The population recruited here, university and NHS staff, is not representative of the general population. There is an overrepresentation of females, which could be accounted for by a combination of factors: female gender bias in internet surveys,^18^ ease of access to the internet, interest in the survey subject matter, and recruiting from predominantly female workforces. However, the patterns of reported drinking and dependence scores are as expected. The gender imbalance is immaterial since calculations are made separately for males and females. Dependence scores varied with different levels of reported substance use: mean LDQ for abstainers of both alcohol and drugs was 0.37, hence abstainers were excluded from the analyses. The CORE-10 and SSQ scores were less affected but participants with illicit drug use had consistently higher CORE-10 scores.

It is not possible to achieve clinically significant improvement if the pre-treatment score is already within the range of the ‘well-functioning’ population. In the clinical sample the SSQ pre-treatment mean is high, indicating that even the clinic population has a general satisfaction with their social circumstances, thus making clinically significant change less likely. Dissatisfaction was most commonly expressed with two SSQ items, employment and finance. There are no overlaps in the confidence intervals for the means of the LDQ and SSQ, compared by gender, between the ‘well-functioning’, pre-treatment and post-treatment populations, suggesting both instruments have the capacity to discriminate between these populations and are sensitive to change.

When applied to the clinical population, the LDQ scores showed most improvement, which might be expected in an addiction treatment service. Individuals with an alcohol problem improved more than those with heroin or other drug problems; drinkers were predominantly male (61.5%), whereas heroin users were predominantly female (55.8%). Where methadone was the main problem drug (typically started as a substitute prescription), change was much less likely than for other substances across all three measures. We looked at outcomes by age group and by episode of treatment - the results are varied and difficult to interpret. The relationship between the LDQ, CORE-10 and SSQ is interesting. Deterioration is more common for CORE-10 and SSQ, which is a well-recognised phenomenon clinically - abstinence or control over substance misuse exposes individuals to the consequences of their substance use and this is commonly expressed as psychological distress and dissatisfaction with social circumstances. These negative experiences reflect the real world and should not necessarily be taken as symptoms of mental illness. Newly abstinent drinkers or, more commonly, drug users may also experience a psychological insecurity which, again, may be expressed in high CORE-10 and low SSQ scores. The precision of short scales is limited and is a tradeoff against the benefits of tools suitable for routine clinical use^19^ - shorter scales need more careful interpretation of results. The combined dimension measures LDQ, CORE-10 and SSQ seem to work well as tools helping better to understand the process of addiction treatment.

The dimensions chosen have consistently been evidenced as important elements of addiction. Dependence is a predictor of treatment outcomes^20,21^ which tends to reduce early in treatment,^22^ and so dependence works well as a feedback tool for practitioners. Equally, social satisfaction is important: the benefits of supportive relationships in long-term recovery from alcohol problems have been well documented^23-26^ and the idea of social support to aid ‘recovery’ is long established.^27,28^ Positive social circumstances play a role in protecting individuals from risks such as depressive affect, psychological distress and stressful life events.^29^ This paper adds to the evidence that the LDQ, CORE-10 and SSQ are appropriate measures and perform well as dimension measures of addiction.
